# Acute Levodopa Challenge in Atypical Parkinsonism: Comprehensive Analysis of Individual Motor Responses

**DOI:** 10.3390/brainsci14100991

**Published:** 2024-09-29

**Authors:** Lan Ye, Sam Sadeghi Sani, Linda Veith Sanches, Lea Farina Magdalena Krey, Florian Wegner, Matthias Höllerhage, Christoph Schrader, Günter Höglinger, Martin Klietz

**Affiliations:** 1 Department of Neurology, Hannover Medical School, 30625 Hannover, Germany; sam.sadeghi-sani@charite.de (S.S.S.); veithsanches.linda@mh-hannover.de (L.V.S.); krey.lea@mh-hannover.de (L.F.M.K.); wegner.florian@mh-hannover.de (F.W.); hoellerhage.matthias@mh-hannover.de (M.H.); schrader.christoph@mh-hannover.de (C.S.); guenter.hoeglinger@med.uni-muenchen.de (G.H.); klietz.martin@mh-hannover.de (M.K.); 2 Department of Neurology, LMU University Hospital, LMU Munich, 80539 Munich, Germany; 3 German Center for Neurodegenerative Diseases (DZNE), 80539 Munich, Germany; 4 Munich Cluster for Systems Neurology (SyNergy), 80539 Munich, Germany

**Keywords:** progressive supranuclear palsy, multiple system atrophy, acute levodopa challenge, treatment

## Abstract

The acute levodopa challenge is widely used to distinguish Parkinson’s disease (PD) from atypical parkinsonian syndromes (APSs) such as multiple system atrophy (MSA) and progressive supranuclear palsy (PSP). In APSs, very few patients present a clinically relevant response to levodopa. The aim of this study was to determine whether patients with atypical parkinsonism benefit from levodopa in any aspect of their multiple motor deficits despite the generally poor response. This retrospective study analyzed individual motor responses to the acute levodopa challenge using the MDS-UPDRS III in 47 PSP, 26 MSA, and 71 PD patients at Hannover Medical School. Despite the generally poor levodopa response in both PSP and MSA patients, bradykinesia and rigidity were the symptoms most notably affected by levodopa in PSP patients, while MSA patients experienced significant improvements in bradykinesia and action tremor. These findings underscore the variability in levodopa response among PSP and MSA patients and highlight the need for personalized treatment approaches in atypical parkinsonism.

## 1. Introduction

Parkinsonism, characterized clinically by rest tremor, bradykinesia, rigidity, and postural instability, has a prevalence of about 0.5% in the German population [1]. Dopamine substitution is one of the major therapeutic strategies for patients with parkinsonism [2,3]. However, the response to dopaminergic therapy differs in parkinsonism. Parkinson’s disease, the most common form of parkinsonism, has a robust-to-excellent response to levodopa and positive dopaminergic responsiveness was proposed as a key supporting feature in established clinical criteria for PD [4]. In contrast, atypical parkinsonian syndromes (APSs), such as multiple system atrophy (MSA) and progressive supranuclear palsy (PSP), show only a poor levodopa response [5]. PSP is the most common APS [6], characterized by ocular motor dysfunction, postural instability, akinesia, and cognitive dysfunction [7], while MSA manifests as rapidly progressive parkinsonism or ataxia in combination with autonomic failure [8].

APSs and PD share a lot of clinical overlaps. It is therefore challenging to differentiate APS from PD clinically [9,10,11]. An acute levodopa challenge has been widely used in clinical practice to prove dopaminergic responsiveness in patients with parkinsonism [12] and is commonly used to distinguish PD from atypical parkinsonism [13]. The efficacy of an acute levodopa challenge is evaluated by the percentage of change in the total Movement Disorder Society–Unified Parkinson’s Disease Rating Scale Part III (MDS-UPDRS III) motor examination score, with a positive response defined as an improvement of more than 30% following levodopa administration [14]. Despite the well-documented overall poor response to levodopa in patients with atypical parkinsonian syndromes, it is currently unknown whether patients with atypical parkinsonism benefit from levodopa in any specific dimension. Although patients with atypical parkinsonism generally exhibit a poor response to levodopa, a small subset of these patients show some positive response [15,16,17]. To address this gap in knowledge, we conducted a retrospective study to explore in detail the response to levodopa in patients with PSP and MSA.

## 2. Methods

### 2.1. Participants

Ethical approval was obtained from the local Ethics Committee at Hannover Medical School No. 8666_Bo_K_2019. All patients gave their written informed consent to participate in this study. Cross-sectional data of PSP, MSA, and PD patients were collected on the wards of the Dept. of Neurology at Hannover Medical School. PSP, MSA, and PD diagnoses were determined by a movement disorder specialist according to the Movement Disorders Society diagnostic criteria for PSP [7], MSA [18], and PD [4]. All patients diagnosed with PSP and MSA who underwent the levodopa challenge in the clinical workup of our biobank were included. The inclusion criterion was the clinically suspected diagnosis of an atypical parkinsonian syndrome. Patients with PD were assessed in the clinical workup of the diagnosis or in the evaluation for non-oral advanced treatment options for PD. We excluded patients with undetermined parkinsonism after careful clinical work. In total, 47 PSP patients, 26 MSA patients, and 71 PD patients who underwent the acute levodopa challenge were retrospectively analyzed. 

### 2.2. Clinical Evaluations

The clinical data obtained include age, disease duration, Hoehn and Yahr stage, 15-item Geriatric Depression Scale (GDS-15) to detect depressive symptoms [19], Montreal Cognitive Assessment (MoCA) to evaluate global cognitive status [20], Movement Disorder Society–Unified Parkinson’s Disease Rating Scale (MDS-UPDRS) scores [21], the Progressive Supranuclear Palsy Rating Scale (PSPRS) to monitor the severity of motor symptoms in PSP patients [22], and the Unified Multiple System Atrophy Rating Scale (UMSARS) parts I and II, which include a historical review and motor examination, respectively, to monitor the severity of motor symptoms in MSA patients [23].

### 2.3. Acute Levodopa Challenge 

An acute levodopa challenge was performed within a standardized test protocol in our department. The day before dopaminergic drug administration, naïve patients, meaning those who had not previously received levodopa, were treated with the peripheral dopamine receptor blocker domperidone (Motilium^®^) at a total daily dose of 40 mg. Then, the patients were examined in the overnight off state. MDS-UPDRS III scores were evaluated before and one hour following levodopa administration. The patients took 200/50 mg levodopa/benserazide immediate-release (Madopar LT^®^) dispersible tablets. The effect of levodopa was categorized based on changes in MDS-UPDRS III scores before and after the treatment with levodopa: less than 20% (none), between 20% and 30% (mild), and more than 30% (good). The detailed components of MDS-UPDRS III were examined, specifically including speech (MDS-UPDRS III 3.1), rigidity (MDS-UPDRS III 3.3), rest tremor (MDS-UPDRS III 3.17, 3.18), other tremors (MDS-UPDRS III 3.15, 3.16), gait and posture (MDS-UPDRS III 3.9, 3.10, 3.11, 3.12, 3.13), and bradykinesia (MDS-UPDRS III 3.2, 3.4, 3.5, 3.6, 3.7, 3.8, 3.14). We calculated the change in the score for each subdomain of the MDS-UPDRS before and after the test to assess the levodopa response for each subdomain. 

### 2.4. Statistics

Data analysis was conducted using R Studio (Version 2023.12.1 + 402). The Shapiro–Wilk test was used to check for normal distribution of the data. The Pearson correlation coefficient was used to explore the linear relationship between normally distributed variables and the Spearman correlation coefficient was used for non-normally distributed variables. An independent *t*-test was used to compare normally distributed variables between two groups. The Mann–Whitney U test was used to compare non-normally distributed variables between two groups. One-way ANOVA followed by the Tukey post hoc test was calculated to compare normally distributed variables among groups, while the Kruskal–Wallis test followed by a pairwise Wilcoxon test was used for non-normally distributed data.

## 3. Results

### 3.1. Demographic Characteristics of PSP and MSA Patients

Forty-seven patients with PSP were included in this study; among them, nineteen were female. The average age of PSP patients was 70.0 ± 8.8 years, with a mean disease duration of 3.8 ± 1.0 years. The Hoehn and Yahr stage was 3.4 ± 0.8, MDS-UPDRS III score on average was 38 ± 16, and the mean PSPRS score was 31.7 ± 13.1. GDS-15 yielded a mean of 4.3 ± 3.3 and the MoCA total score averaged at 22.7 ± 5.2 (Table 1). Among these PSP patients, 97.9% had Richardson syndrome (PSP-RS), while only 2.1% had progressive gait freezing (PSP-PGF). Other types of PSP were not observed in this study (Table 2). 

Twenty-six patients, among them eighteen females, diagnosed with MSA were included in this study. They exhibited an average age of 62.7 ± 9.5 years, and a mean disease duration of 4.6 ± 2.1 years. They had an MDS-UPDRS III score of 49.1 ± 21.8. The UMSARS I and II sum score revealed an average of 47.2 ± 15.4. GDS-15 evaluation resulted in a mean of 6.6 ± 3.3, and the MoCA total score averaged at 24.2 ± 5.8 (Table 1). Of these MSA patients, 40.9% were diagnosed with multiple system atrophy, cerebellar type (MSA-C), while 59.1% were diagnosed with multiple system atrophy, parkinsonian type (MSA-P) (Table 2).

### 3.2. Detailed Analysis of Levodopa Response in PSP and MSA Patients

**Figure 1:**
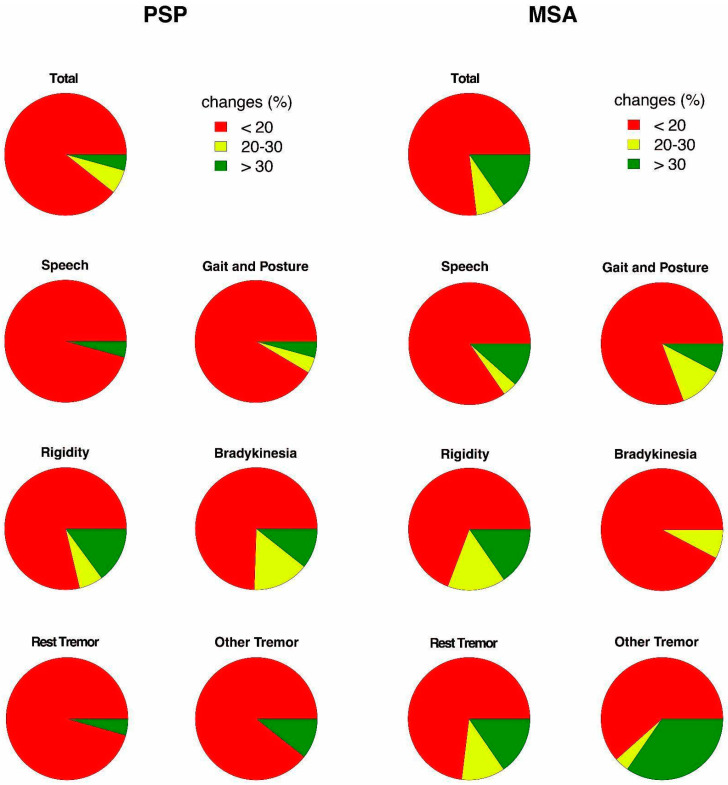
The percentage of patients with PSP and MSA exhibiting different levels of change in UPDRS III scores following an acute levodopa challenge. The patients were categorized into three groups based on the percentage change in their UPDRS III scores: less than 20%, between 20% and 30%, and more than 30%. The left two columns illustrate PSP patients, while the right two columns represent MSA patients.

**Figure 2:**
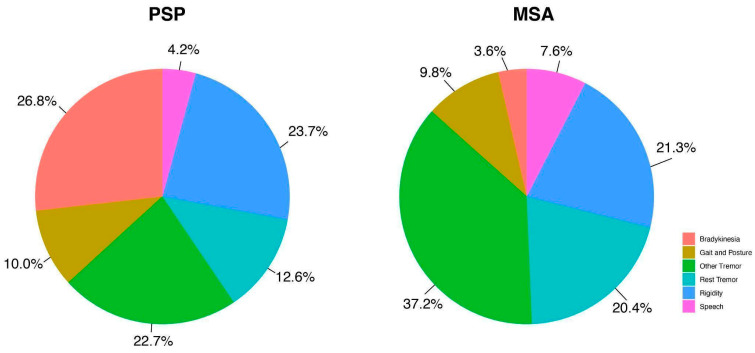
Distribution of symptom change in patients with PSP and MSA following the acute levodopa challenge. Each color segment in the pie charts represents the relative contribution of each symptom category to the total observed improvement across all six categories. The percentages were calculated by using each symptom’s improvement percentage relative to the combined improvement percentage across all symptoms. The left pie chart illustrates PSP patients, while the right chart represents MSA patients.

Of the patients with PSP, 89.4% showed no significant change in the total MDS-UPDRS III score following treatment, and only 4.2% of patients showed a good improvement (Figure 1). However, PSP patients exhibited more changes in ‘rigidity’, with 14.9% of patients showing a good response, followed by ‘bradykinesia’ (10.6% of patients with a good response). Conversely, nearly no PSP patients showed improved ‘speech’ under administration of levodopa (95.7% of patients with no change). Very few patients showed reduced ‘rest tremor’ (93.6% of patients showing no changes) or improved gait (91.5% of patients with no change). Regarding the distribution of the responsive symptoms (Figure 2), ‘bradykinesia’ (26.8%) was the symptom that responded most to levodopa, followed by ‘rigidity’ (23.7%), ‘tremors other than rest tremor’ (22.7%), ‘rest tremor’ (12.6%), and ‘gait and posture’ (10.0%). ‘Speech’ showed the least response, with only 4.2% of patients exhibiting improvement. The Kruskal–Wallis test showed a significant difference among these groups (*p* < 0.0001).

Of the MSA patients, 76.9% had no change in the total MDS-UPDRS III score following the acute levodopa challenge. Among all the six sub-items, ‘tremors other than rest tremor’ showed the best improvement, as 34.6% of MSA patients exhibited a good response in that domain. Among MSA patients, 15.4% also improved in ‘rest tremor’ and ‘rigidity’. ‘Bradykinesia’ showed the worst response, with 92.3% of MSA patients showing no improvement in the acute levodopa challenge. As shown in Figure 2, the most responsive symptom was ‘tremors other than rest tremor’ (37.2%), followed by ‘rigidity’ (21.3%), ‘rest tremor’ (20.4%), ‘gait and posture’ (9.8%), ‘speech’ (7.6%), and ‘bradykinesia’ (3.6%). A statistical difference among these groups was also revealed by the Kruskal–Wallis test (*p* < 0.05).

### 3.3. Comparison of the Response to Levodopa among PD, MSA, and PSP Patients

**Figure 3:**
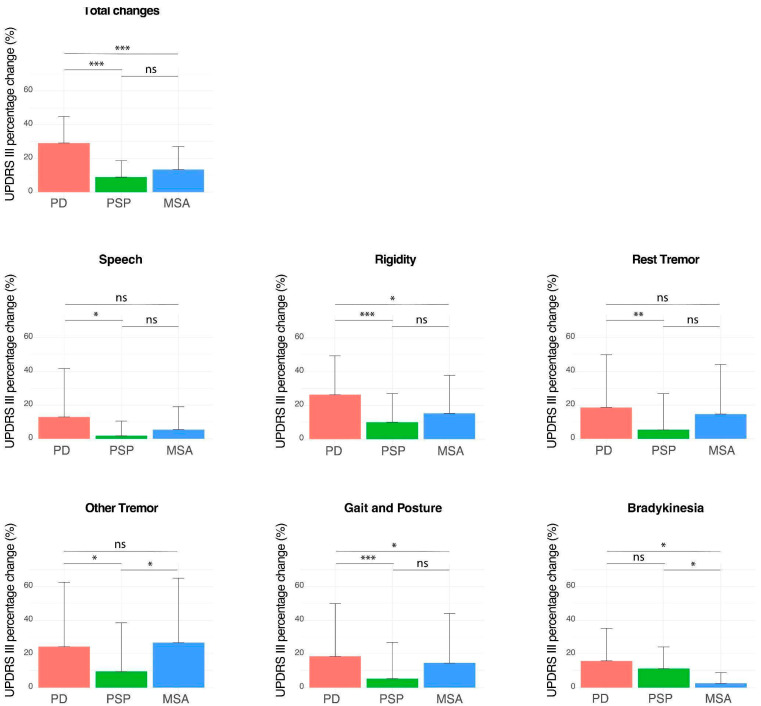
Comparison of the change in MDS-UPDRS III scores following acute levodopa challenge among PD, PSP, and MSA patients. The percentage changes in MDS-UPDRS III scores are shown, represented as mean ± standard deviation. *** *p* < 0.001; ** *p* < 0.01; * *p* < 0.05; ns: not significant.

As illustrated in Figure 3 (the concrete data are presented in Table S1), the statistical analysis showed that PD patients exhibited a significantly higher response, as measured by percentage of change in MDS-UPDRS III total score, following the levodopa challenge compared to PSP (PD: 28.5% ± 17.5% vs. PSP: 8.1% ± 10.8%; ANOVA with Tukey post hoc test; *p* < 0.0001) and MSA patients (PD: 28.5% ± 17.5% vs. MSA: 11.8% ± 16.2%; ANOVA with Tukey post hoc test; *p* < 0.0001). There was no significant difference in the percentage of change in MDS-UPDRS III total score between the PSP and MSA groups (PSP: 8.1% ± 10.8% vs. MSA: 11.8% ± 16.2%; ANOVA with Tukey post hoc test; *p* > 0.05).

We compared the response to levodopa between MSA-C and MSA-P patients. It was shown that MSA-P patients had a significantly better response than MSA-C patients (MSA-P vs. MSA-C: 16% ± 14.2% vs. 3.0% ± 4.9%; *t*-test; *p* < 0.01). A statistical analysis to compare the response between PSP-RS and PSP variants (including PSP-PFG and PSP-CBS) was not possible due to the significant difference in the number of patients in the two groups.

Regarding bradykinesia, this symptom showed the best response to levodopa in PSP patients; there was no significant difference in levodopa response between PSP and PD patients (*p* > 0.05). However, both the PSP and PD patients had a significantly better response of bradykinesia to levodopa compared to the MSA group (*p* < 0.05). Similarly, ‘tremor, other than rest tremor’, the symptom with the best improvement in MSA patients, showed an equally good response compared to PD patients (Kruskal–Wallis test followed by pairwise Wilcoxon test; *p* > 0.05), and a significantly better response than in PSP patients (Kruskal–Wallis test followed by pairwise Wilcoxon test; *p* < 0.05). Rigidity, which was the second most responsive symptom in both MSA and PSP patients, exhibited a significantly weaker improvement in both groups compared to PD patients. However, when comparing the response of rigidity between PSP and MSA patients, no significant difference was observed (Kruskal–Wallis test followed by pairwise Wilcoxon test; *p* > 0.05).

No difference in response to acute levodopa challenge was observed between males and females, including the total score and all subscores, in either the MSA or PSP groups (Table S2).

### 3.4. Correlation of Clinical Variables Related to the Response of Acute Levodopa Challenge

To identify factors contributing to the response to the acute levodopa challenge, linear regression analysis was performed to explore the relationship between the change in MDS-UPDRS III score and the clinical symptoms of PSP/MSA patients. These variables included the age of the patients at the time of the levodopa test and at the first clinical manifestation, the duration of the disease, the Hoehn and Yahr score, the PSPRS/UMSARS, the total MoCA score, and the MoCA executive function score. As shown in Table 3, none of the comparisons revealed any significant correlation among MSA patients or PSP patients.

Patients were further divided into two groups according to their response to the acute levodopa challenge: no response and mild-to-good response. No difference was observed between patients with no response to levodopa and patients with a mild-to-good response to levodopa in the above parameter (Table S3, Mann–Whitney U test, *p* > 0.05).

## 4. Discussion

The summarized findings of this study are as follows: (1) The majority of PSP and MSA patients demonstrated a generally poor response to acute levodopa challenge. (2) In PSP patients, bradykinesia and rigidity were the symptoms most notably affected by levodopa, while MSA patients experienced significant improvements in bradykinesia and action tremor. (3) No significant correlations were found between the change in MDS-UPDRS III score following the acute levodopa challenge and various variables, such as age, disease duration, and clinical scores, indicating that these factors do not predict levodopa response in PSP and MSA.

The limited efficacy of levodopa in patients with PSP and MSA in this study aligns with previous studies reporting poor levodopa responsiveness as a hallmark of these diseases [5]. To exclude the possibility of a technical deficit affecting the poor levodopa response in patients with atypical parkinsonism, we compared their results with those of PD patients, following the same protocol and conducted by the same personnel. The comparison showed that PD patients had a better levodopa responsiveness, indicating that the poor response in atypical parkinsonism patients was not due to an examination technique.

Upon further examination, PSP patients showed significant improvement in rigidity and bradykinesia from levodopa therapy. This result is consistent with reports that levodopa can specifically provide some benefit for bradykinesia and rigidity in PSP patients [24]. On the other hand, rigidity and action tremor responded more positively to the acute levodopa challenge in MSA patients. Unlike rest tremor, which is found in more than 70% of PD patients [25,26], action tremor, especially postural tremor, is common in MSA patients and is a supportive feature of MSA [18]. Notably, in PSP, there are distinct phenotypes according to the dominant symptoms, such as Richardson syndrome (PSP-RS), ocular motor dysfunction (PSP-OM), PSP–postural instability (PSP-PI), and PSP–parkinsonism (PSP-P) [7]. MSA patients are stratified into two phenotypes: patients with predominant parkinsonism (MSA-P) or with predominant cerebellar syndrome (MSA-C) [8]. Based on our observations, we hypothesize that PSP patients with predominant bradykinesia and rigidity and MSA patients with predominant rigidity or action tremor may benefit more from the levodopa therapy. However, further studies are required to validate this hypothesis and to refine treatment strategies accordingly. Particularly, it would be very interesting to observe the levodopa response of PSP-P patients, because this may help in the differential diagnosis of parkinsonism.

In our study, only 200 mg levodopa was used for the acute levodopa challenge. A study involving pharmacodynamic monitoring of levodopa found that the magnitude and overall extent of the levodopa tapping effect were significantly reduced in patients with MSA compared to PD, although their levodopa pharmacokinetics did not differ significantly [27]. This indicates that higher daily doses of levodopa and/or longer treatment durations could be more effective. The North American MSA Study Group suggests that levodopa responsiveness should be tested over a 3-month period with escalating doses up to at least 1000 mg/day [5]. In a study of PSP patients, intrajejunal infusion therapy of high doses of levodopa was found to be effective [28]. Thus, for patients who do not benefit from the acute levodopa challenge, a chronic therapy approach with slow dose increments could be considered. However, caution should be taken due to the potential for worse side effects from higher drug dosages, which can be intensified through altered gastric motility [27]. It was also reported that patients with atypical parkinsonism may experience more frequent and severe side effects from levodopa therapy [29].

Identifying patients who are more likely to benefit from levodopa therapy than suffer side effects from it is important. Unfortunately, we did not find any potential confounding factors that influence the response to levodopa in patients with PSP or MSA, such as disease duration, disease severity, global cognitive status, executive function, and mood status in response to levodopa.

Different patterns of degeneration of dopaminergic neurons in the brainstem and striatum were observed in PD, PSP, and MSA patients [30]. Reduced postsynaptic dopamine receptors were observed in atypical parkinsonism compared to PD patients [31]. Despite these distinct variations in the pathogenesis of the dopaminergic system, which may contribute to differing levodopa responsiveness, the reasons for varying levodopa responses among PD, PSP, and MSA, as well as the variability in responsiveness within the APS groups and across different symptoms, remain unclear. Further research is needed to elucidate the precise mechanisms underlying these differences and to identify biomarkers that could predict individual responses to levodopa treatment more accurately.

## 5. Limitations

There are several limitations of this study. The relatively small sample size of PSP and MSA patients limits the generalizability of the findings. Moreover, we did not capture all dimensions of motor and particularly non-motor symptoms that could be affected by levodopa treatment, and this study did not consider the influence of comorbidities in PD, PSP, and MSA [32,33,34]. The comparison among PD, PSP, and MSA groups was not age matched. The investigator was not blind to the patients’ complete history and diagnoses, which may have introduced bias. The diagnosis was purely clinical, not confirmed neuropathologically. Further, this study only reports the results of an acute levodopa challenge. However, improvement in chronic levodopa treatment cannot be ruled out, which must be addressed in future studies.

## 6. Conclusions

In conclusion, the generally poor response to levodopa in MSA and PSP patients, despite the symptom-specific improvements observed, underscores the need for a nuanced understanding of these complex disorders. By continuing to explore the diverse factors influencing treatment response and integrating this knowledge into clinical practice, we can move closer to developing personalized and more effective therapeutic strategies for individuals with atypical parkinsonian syndromes.

## Figures and Tables

**Table 1:** Demographic characteristics of PSP (*n* = 47) and MSA (*n* = 26) patients.

	PSP				MSA			
	Mean ± SD	Med	Min	Max	Mean ± SD	Med	Min	Max
Age (years)	70.0 ± 8.8	71.5	48	89	62.9 ± 9.5	62.5	45	82
Disease duration (years)	3.8 ± 1.0	3.7	2	9	4.6 ± 2.1	4.0	2	9
H & Y stage	3.4 ± 0.8	3.0	1	5	3.5 ± 0.8	3.5	2	5
MDS-UPDRS III	38 ± 16	37.0	8	77	49.1 ± 21.8	44.5	17	91
GDS- 15	4.3 ± 3.3	4.0	1	15	6.6 ± 3.3	7	1	12
MoCA	22.7 ±5.2	23.5	8	29	24.2 ± 5.8	26	4	30
PSPRS	31.7 ± 13.1	30.5	16	80	-	-	-	-
UMSARS I and II	-	-	-	-	47.2 ± 15.4	41.0	27	75

Abbreviations: GDS, Geriatric Depression Scale; H & Y stage, Hoehn and Yahr stage; MDS-UPDRS, Movement Disorder Society–Unified Parkinson’s Disease Rating Scale; Max, maximum; Med, median; Min, minimum; MoCA, Montreal Cognitive Assessment; MSA, multiple system atrophy; PSP, progressive supranuclear palsy; PSPRS, Progressive Supranuclear Palsy Rating Scale; SD, standard deviation; UMSARS I and II, Unified Multiple System Atrophy Rating Scale part I and part II.

**Table 2:** Distribution of the phenotype among MSA and PSP patients.

Phenotypes	MSA-C	MSA-P	PSP-PGF	PSP-RS
Percentages	40.9%	59.1%	2.1%	97.9%

Abbreviations: MSA-C: multiple system atrophy, cerebellar type; MSA-P: multiple system atrophy, parkinsonism type; PSP-PGF: progressive supranuclear palsy with progressive gait freezing; PSP-RS: progressive supranuclear palsy with Richardson syndrome. Four MSA patients presented with parkinsonian and cerebellar signs in an equal proportion and, as a result, could not be classified as MSA-P or MSA-C and were not included in the calculation of the distribution.

**Table 3:** Correlation of clinical variables related to the response of acute levodopa challenge among PSP and MSA patients.

	PSP		MSA	
	*R*	*p*	*R*	*p*
Age in years	0.05 ^&^	0.74	−0.30 ^$^	0.14
Disease duration in years	−0.28 ^$^	0.09	0.01 ^$^	0.97
MDS-UPDRS III	−0.07 ^$^	0.66	0.07 ^$^	0.74
PSPRS	−0.15 ^$^	0.37	-	-
UMSARS I and II	-	-	−0.02 ^&^	0.93
MoCA total	−0.26 ^$^	0.11	0.13 ^$^	0.55
MoCA executive	−0.25 ^$^	0.13	0.31 ^$^	0.13
H and Y stage	−0.06 ^$^	0.72	0.24 ^$^	0.27

Abbreviations: GDS, Geriatric Depression Scale; H and Y stage, Hoehn and Yahr stage; MDS-UPDRS III, Movement Disorder Society–Unified Parkinson’s Disease Rating Scale part III; MoCA, Montreal Cognitive Assessment; MSA, multiple system atrophy; PSP, progressive supranuclear palsy; PSPRS, Progressive Supranuclear Palsy Rating Scale; *R*, correlation coefficient; UMSARS I and II, Unified Multiple System Atrophy Rating Scale part I and part II. ^&^ Pearson correlation coefficient; ^$^ Spearman correlation coefficient.

## Data Availability

The data supporting the findings of this study are available from the corresponding author Dr. Lan Ye upon reasonable request. They are not publicly available because of ethical concerns.

## Supplementary Materials

The following supporting information can be downloaded at: https://www.mdpi.com/article/10.3390/brainsci14100991/s1, Table S1. Comparison of the change in MDS-UPDRS III scores following acute levodopa challenge among PD, PSP and MSA patients. Table S2. Gender difference between PSP and MSA patients. Table S3. Comparison of clinical variables between no response and mild good response patients with PSP and MSA.
