# Supplementary material for: Acute Levodopa Challenge in Atypical Parkinsonism: Comprehensive Analysis of Individual Motor Responses
Source: Brain Sci. 2024 Sep 29;14(10):991. doi: 10.3390/brainsci14100991 (PMC11506334; doi:10.3390/brainsci14100991)
Supplement: Supplementary file 1 [file brainsci-14-00991-s001.zip › brainsci-3204652-supplementary.pdf]

Table S1. Comparison of the change in MDS-UPDRS III scores following acute levodopa challenge among PD, PSP and MSA patients.

	PD	PSP	MSA	post-hoc results
**Total changes (%)**	29.1±15.8	8.9±9.8	13.4±13.6	***PD> MSA<br>***PD> PSP<br>nsPDvs MSA
**Speech (%)**	12.9±28.8	1.9±8.7	5.5±13.5	nsPD vs MSA<br>*PD > PSP<br>nsPD vs MSA
**Rigidity (%)**	26.3±23.1	10.0±17.0	15.3±22.5	*PD > MSA<br>***PD > PSP<br>nsPSP vs MSA
**Rest Tremor (%)**	18.6±31.3	5.3±21.5	14.6±29.4	nsPD vs MSA<br>**PD > PSP<br>nsPSP vs MSA
**Other Tremor (%)**	24.3±38.2	9.6±28.8	26.7±38.3	nsPD vs MSA<br>*PD > PSP<br>*PSP < MSA
**Gait and Posture (%)**	18.6±31.3	5.3±21.5	14.6±29.4	*PD > MSA<br>***PD > PSP<br>nsPSP vs MSA
**Bradykinesia (%)**	15.8±19.3	11.3±12.8	2.6±6.3	*PD > MSA<br>nsPD vs PSP<br>*PSP > MSA

The values are presented as mean ± SD. One-way ANOVA followed by Tukey post hoc test was calculated to compare "Total changes" among groups, while Kruskal-Wallis test followed by pairwise Wilcoxon test was used for the other parameters. ***$p < 0.001$; **$p < 0.01$; *$p < 0.05$; ns$p$: not significant.

Table S2. Gender difference between PSP and MSA patients.

	PSP			MSA		
	male	female	*p*	male	female	*p*
total score	7.4±9.9	11.1±9.7	0.08	11.8±14.3	14.0±13.6	0.49
Speech	1.8±9.5	1.7±7.6	0.83	4.13±11.67	6.0±14.5	0.82
Gait and Posture	3.6±9.5	5.0±9.1	0.35	11.75±14.25	11.8±14.3	0.37
Rigidity	6.9±14.6	14.6±19.5	0.08	12.63±11.67	16.5±14.8	1
Bradykinesia	11.8±14.3	11.8±14.3	0.50	4.0±14.3	2.0±13.6	0.60
Rest Tremor	1.8±9.5	10.5±31.5	0.33	6.3±14.3	8.3±13.6	0.32
Other Tremor	8.9±27.4	10.5±31.5	1	39.4±45.5	25.1±36.1	1

Abbreviations: MSA, Multiple System Atrophy; PSP, Progressive Supranuclear Palsy; PSPRS, Progressive Supranuclear Palsy Rating Scale. Mann-Whitney U Test was used.

Table S3. Comparison of clinical variables between no response and mild good response patients with PSP and MSA

	PSP			MSA		
	**no response**	**mild-good response**	***p***	**no response**	**mild-good response**	***p***
**Age in years**	73.7 ±8.3	65.8±10.9	0.10	62.7±9.7	63.3±9.8	0.93
**Disease duration in years**	3.9±1.4	3.5±1.0	0.90	4.3±1.6	5.3±2.5	0.38
**MDS-UPDRS III**	40.5±17.8	31.0±11.1	0.25	48.3±22.0	51.7±23.0	1
**PSPRS**	30.2±10.5	43.3±25.9	0.30	-	-	-
**UMSARS I & II**	-	-	-	48.1±16.7	42.2±11.2	0.70
**MoCA Total**	20.5±8.2	23.0±4.5	0.67	24.5±5.8	23.4±6.0	0.73
**MoCA Executive**	3.3±1.1	3.3±1.2	0.92	3.6±1.7	4.0±1.7	0.47
**H&Y stage**	3.4±0.8	3.3±0.6	0.74	3.6±0.8	3.4±0.5	0.49

Abbreviations: H&Y stage, Hoehn and Yahr stage; MDS-UPDRS III, Movement Disorder Society Unified Parkinson's Disease Rating Scale part III; MoCA, Montreal-Cognitive-Assessment; MSA, Multiple System Atrophy; PSP, Progressive Supranuclear Palsy; PSPRS, Progressive Supranuclear Palsy Rating Scale; UMSARS I & II, Unified Multiple System Atrophy Rating Scale part I and part II. Mann-Whitney U Test was used.
